# Clusters of heterogeneity of tuberculosis-HIV coinfection in Brazil: a geospatial study

**DOI:** 10.11606/s1518-8787.2024058005531

**Published:** 2024-03-27

**Authors:** Lucas Vinícius de Lima, Gabriel Pavinati, Pedro Augusto Bossonario, Aline Aparecida Monroe, Daniele Maria Pelissari, Kleydson Bonfim Andrade Alves, Gabriela Tavares Magnabosco

**Affiliations:** I Universidade Estadual de Maringá Programa de Pós-Graduação em Enfermagem Maringá PR Brazil Universidade Estadual de Maringá. Programa de Pós-Graduação em Enfermagem. Maringá, PR, Brazil; II Universidade de São Paulo Programa de Pós-Graduação Enfermagem em Saúde Pública Ribeirão Preto SP Brazil Universidade de São Paulo. Programa de Pós-Graduação Enfermagem em Saúde Pública. Ribeirão Preto, SP, Brazil; III Brazilian Ministry of Health Departamento de HIV/aids, Tuberculose, Hepatites Virais e Infecções Sexualmente Transmissíveis Brasília DF Brazil Brazilian Ministry of Health. Departamento de HIV/aids, Tuberculose, Hepatites Virais e Infecções Sexualmente Transmissíveis. Brasília, DF, Brazil; IV Pan American Health Organization Department of Communicable Diseases and Environmental Determinants of Health Brasília DF Brazil Pan American Health Organization. Department of Communicable Diseases and Environmental Determinants of Health. Brasília, DF, Brazil

**Keywords:** HIV, Tuberculosis, Coinfection, Spatial analysis

## Abstract

**OBJECTIVE:**

To analyze the geospatialization of tuberculosis-HIV coinfection in Brazil, from 2010 to 2021, and the correlation with socioeconomic, housing, and health indicators.

**METHODS:**

An ecological study of Brazilian municipalities and states, with data from HIV and tuberculosis information systems, previously reported by the Ministry of Health. The crude and smoothed coefficients were calculated by the local empirical Bayesian method of incidence of coinfection per 100,000 inhabitants in the population aged between 18 and 59 years. Univariate (identification of clusters) and bivariate (correlation with 20 indicators) Moran’s indices were used.

**RESULTS:**

A total of 122,223 cases of coinfection were registered in Brazil from 2010 to 2021, with a mean coefficient of 8.30/100,000. The South (11.44/100,000) and North (9.93/100,000) regions concentrated the highest burden of infections. The coefficients dropped in Brazil, in all regions, in the years of covid-19 (2020 and 2021). The highest coefficients were observed in the municipalities of the states of Rio Grande do Sul, Mato Grosso do Sul, and Amazonas, with high-high clusters in the capitals, border regions, coast of the country. The municipalities belonging to the states of Minas Gerais, Bahia, Paraná, and Piauí showed low-low clusters. There was a direct correlation with human development indices and aids rates, as well as an indirect correlation with the proportion of poor or of those vulnerable to poverty and the Gini index.

**CONCLUSIONS:**

The spatial analysis of tuberculosis-HIV coinfection showed heterogeneity in the Brazilian territory and constant behavior throughout the period, revealing clusters with high-burden municipalities, especially in large urban centers and in states with a high occurrence of HIV and/or tuberculosis. These findings, in addition to alerting to the effects of the covid-19 pandemic, can incorporate strategic planning for the control of coinfection, aiming to eliminate these infections as public health problems by 2030.

## INTRODUCTION

Tuberculosis and human immunodeficiency virus (HIV) infection persist as public health challenges, particularly for health systems and services, given the complexity resulting from the possible overlap of these conditions, as well as the existence of structural, organizational, socioeconomic, cultural, and behavioral aspects that touch on their occurrence^1^.

The relationship between tuberculosis and HIV is strong, especially due to the greater susceptibility of people affected by the virus to developing the active form and unfavorable outcomes of tuberculosis^1,3,4^, making them a priority group for prevention, treatment, and control actions. In 2021, 6.4 million cases of tuberculosis were registered worldwide, 6.7% of which were in people living with HIV and, of these, 187,000 resulted in death, reiterating the high mortality of these infections^2^.

Ending the tuberculosis and HIV epidemics, as well as the concomitance of infections, is among the Sustainable Development Goals (SDGs) signed by the United Nations (UN)^1,5^. These objectives are even more important in the 30 countries with a high burden of coinfection listed by the World Health Organization (WHO), among which Brazil stands out, with a proportion of 10.3% of HIV among tuberculosis cases in 2019^1,2,6^.

Given the continental dimensions of the country, the behavior of coinfection among Brazilian territories shows discrepancies, due to social and programmatic factors^6^. The disease mainly affects people who experience situations of vulnerability and precarious living conditions, engage in health risk practices, and/or lack resources to access strategies for the prevention, diagnosis, and treatment for HIV and tuberculosis^7,9^.

Despite the high morbidity and mortality of tuberculosis-HIV coinfection, conducting studies on the epidemiological scenario in Brazil presents some difficulties^7,8,10,11^, possibly due to the fragility of the record of double infection, which requires a relationship between both databases to qualify the notification^6^. Therefore, studies on the influence of geopolitical and social territory on the occurrence of coinfection are needed.

In this sense, spatial studies have been essential to surveil measures of incidence and morbidity and mortality^7,12^, especially in the interface with infectious and contagious conditions that, historically, are related to disparities in the Brazilian territory^13^. That said, this study aimed to analyze the geospatialization of the incidence of tuberculosis-HIV coinfection in Brazil, from 2010 to 2021, and the correlation with socioeconomic, housing, and health indicators.

## METHODS

This is a geospatial ecological study, whose analytical units were the geographic regions, the federative units (FU), and the Brazilian municipalities. In its political-administrative division, Brazil includes 5,568 municipalities—plus the Federal District and the State District of Fernando de Noronha—which are organized into 27 states. These, in turn, are arranged in five geographic regions: North, Northeast, South, Southeast, and Midwest^14^.

The HIV and tuberculosis surveillance is guided by programs linked to the Health and Environment Surveillance Secretariat of the Ministry of Health (SVSA/MS). Based on national recommendations, the states and municipalities organize themselves to offer actions and services aimed at the management and control of tuberculosis and HIV. Health information, in turn, is centralized in the national notification and monitoring systems.

To delimit the cases of tuberculosis-HIV coinfection, data from the *Sistema de Informação de Agravos de Notificação* (SINAN – Notifiable Diseases Information System), the *Sistema de Controle de Exames Laboratoriais da Rede Nacional de Contagem de Linfócitos CD4+/CD8+ e Carga Viral do HIV* (SISCEL – Laboratory Test Control System of the National CD4+/CD8+ Lymphocyte Count and HIV Viral Load Network), the *Sistema de Controle Logístico de Medicamentos* (SICLOM – Drug Logistics Control System), and the *Sistema de Informação sobre Mortalidade* (SIM – Mortality Information System) were considered and connected probabilistically in the Reclink^®^ software.

The database, according to the methodological description in the epidemiological bulletin of tuberculosis-HIV coinfection published in 2021^6^, was requested from the Ministry of Health, via the *Sistema Eletrônico de Informações ao Cidadão* (e-SIC – Electronic Citizen Information System), under protocol No. 25072.039887/2022-27. Three probabilistic relationships were carried out: the first between the HIV bases, the second between the tuberculosis bases, and the third between the products of the first two stages^6^.

The population data from the estimation study were accessed from the *Departamento de Informática do Sistema Único de Saúde* (DATASUS – Department of Informatics of the Unified Health System)^15^. In addition, the socioeconomic, housing, and health indicators from the *Sistema Nacional de Informações sobre Saneamento* (SNIS – National Sanitation Information System), the *Pesquisa Nacional por Amostra de Domicílios* (PNAD – National Household Sample Survey), and DATASUS for 2017 were consulted in the Atlas of Human Development^16^ (Box).

**Box:** Development indicators considered for correlation with the crude tuberculosis-HIV coinfection incidence coefficients in the federated units of Brazil.

Indicator	Code	Source
Socioeconomic		
Human development index	V1	PNAD (2017)
Human development index – income	V2	PNAD (2017)
Human development index – longevity	V3	PNAD (2017)
Human development index – education	V4	PNAD (2017)
Percentage of people vulnerable to poverty	V5	PNAD (2017)
Percentage of poor people	V6	PNAD (2017)
Gini Index	V7	PNAD (2017)
*Per capita* income	V8	PNAD (2017)
Percentage of people of 18 years of age or older with complete primary education	V9	PNAD (2017)
Illiteracy rate of people of 18 years of age and older	V10	PNAD (2017)
Mean years of schooling	V11	PNAD (2017)
Housing		
Percentage of households connected to the water supply network	V12	SNIS (2017)
Percentage of households connected to the sewage system	V13	SNIS (2017)
Existence of selective collection	V14	SNIS (2017)
Percentage of hospitalizations related to inadequate environmental sanitation	V15	DATASUS (2017
Sanitary		
Life expectancy at birth	V16	PNAD (2017)
Hospitalizations rates due to primary care sensitive conditions	V17	DATASUS (2017)
Percentage of people covered by supplemental health insurance	V18	DATASUS (2017)
Crude mortality rate	V19	DATASUS (2017)
Aids incidence rate	V20	DATASUS (2017)

PNAD: *Pesquisa Nacional por Amostra de Domicílios* (National Household Sample Survey); SNIS: *Sistema Nacional de Informações sobre Saneamento* (National Sanitation Information System); DATASUS: *Departamento de Informática do Sistema Único de Saúde* (Department of Informatics of the Unified Health System).

Coinfection cases were defined as people whose tuberculosis record on SINAN (reported as a “new case”), regardless of the clinical form, had the variable “HIV” marked as “positive” or the variable “aids disease” marked as “yes”; or those reported for tuberculosis on SINAN or SIM without these variables being filled in, but who had been diagnosed in the HIV databases, who had some result in SISCEL or who had dispensing in SICLOM^6^.

People aged 18 to 59 years were included since they correspond to most of the cases of coinfection registered in the country (±91.88%)^6^. The variable referring to the municipality of residence was also considered. However, note that 68 people had this information ignored at the time of notification. Thus, the georeferencing of these cases was performed by the municipality of notification, representing a proxy variable of the residence.

The time frame from 2010 to 2021 included the following periods: prior to the incorporation of the rapid molecular test for tuberculosis (2010–2013); prior to the publication of the tuberculosis infection surveillance protocol (2014–2017); prior to the covid-19 pandemic (2018–2019); and concomitant with covid-19 (2020–2021). These interventions may have influenced the prevention, detection, and/or notification of tuberculosis in people with HIV and, therefore, modified the spatial behavior.

The data were tabulated according to the aforementioned quadrennium and biennium. Initially, for the spatial distribution of tuberculosis-HIV coinfection cases, the crude incidence coefficients for each municipality, state, and region of the country were calculated based on the ratio of new cases by the resident population in the same period, age, and location. The result was multiplied by 100,000 inhabitants.

Given the existence of sparsely populated municipalities, the observation of cases could lead to low representativeness of the coefficients, causing distortion and high variability. Thus, smoothing was carried out using the local empirical Bayesian method, aiming to reduce instabilities by generating coefficients by weighting the values of the borders, defined by the contiguity of the first-order queen type to restrict random fluctuation^17^.

From this, maps were built for the spatial distribution, organized from the natural breaks in a scale of gray tones with light and dark colors, representing the minimum and maximum coefficients respectively. This classification ensured the homogeneity and heterogeneity of the data, by “breaking” the intervals, minimizing their variation in the same group and maximizing the variation of values between groups^18^.

Moran’s statistic was used in the smoothed coefficients, subdivided into global (I_MG_) and local (I_ML_) Moran’s index. Univariate I_MG_ was calculated with the respective pseudosignificance test, with 999 permutations (p < 0.05). The I_MG_ tests the spatial dependence hypothesis by providing a measure ranging from −1.00 to +1.00. Values close to zero indicate randomness and values close to one indicate direct (+) or inverse (−) spatial autocorrelation^19^.

Once the significance of the I_MG_ was proven, the univariate I_LM_ was calculated, a type of local indicator of spatial association to identify clusters that influence the I_MG_, namely: high-high (HH), areas and neighboring areas with high coefficients; low-low (LL), areas and neighboring areas with low coefficients; low-high (LH), areas with low and neighboring areas with high coefficients; high-low (HL), areas with high and neighboring areas with low coefficients; and non-significant (NS), areas with no spatial trend^20^.

Aggregated data at the state level were used to associate the incidence coefficients of tuberculosis-HIV coinfection with the indicators shown in the Box, given the unavailability of recent indicators for municipalities in Brazil at the time this study was conducted. Thus, the arithmetic means of the crude incidence coefficients of each state were considered as the dependent variable and the indicators as the independent variables.

These mean coefficients were turned into logarithms to approximate a normal distribution. Then, a Spearman correlation matrix was constructed for the bivariate analysis, in which the variables were unable to present a significant association (p > 0.50), making it impossible to construct a regression model. Due to this, Moran’s bivariate analysis was used, which seeks to verify the association between two variables based on the values in neighboring regions^20^.

In Moran’s bivariate analysis, the clusters were classified as: HH, areas with high coefficients and indicators; LL, areas with low coefficients and indicators; LH, areas with low coefficients and high indicators; HL, areas with high coefficients and low indicators; and NS, areas with no association^20^. The analyses were performed in a Microsoft Excel^®^ 2016 spreadsheet and in GeoDa^®^ (version 1.20.0) and QGIS^®^ (version 2.36.3) software.

To broaden the depth of the study, the vulnerability framework was used, which has three inseparable and interdependent planes, represented by individual, social, and programmatic aspects, which influence the health-disease process. Therefore, the concept of risk is expanded, assuming that illness considers situations associated with socioeconomic, institutional, and geopolitical contexts^21^.

In accordance with the ethical guidelines, recommended by Resolution No. 466/2012 of the National Health Council, this research was approved by the Research Ethics Committee of the Universidade Estadual de Maringá, under opinion No. 5.721.740/2022 and certificate of presentation for ethical appreciation No. 63981922.6.0000.0104. The waiver of the free and informed consent form was requested due to the use of secondary data.

## RESULTS

**Figure 1:**
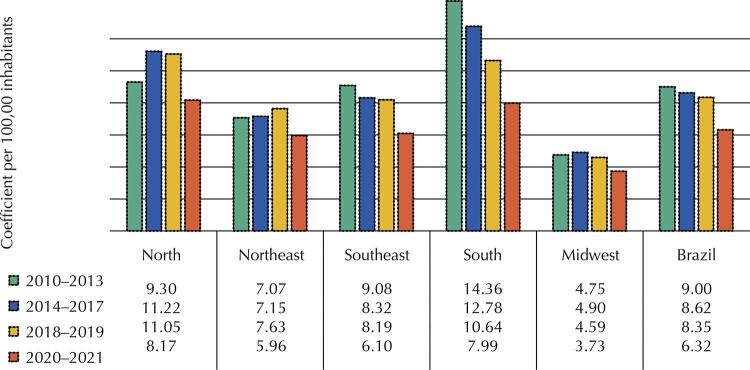
Crude coefficient for the incidence of tuberculosis-HIV coinfection among people aged 18 to 59 years, per 100,00 inhabitants, in Brazilian regions, from 2010 to 2021.

A total of 122,223 cases of tuberculosis-HIV co-infection were reported in Brazil among people aged 18 to 59 years, from 2010 to 2021, with a mean coefficient of 8.30/100,000. The South and North regions concentrated the highest number of cases. The North showed an increase between 2014 and 2017, whereas the South and Southeast showed a reduction over the years. The incidence of coinfection decreased in the country and its macro-regions between 2020 and 2021 (Figure 1).

**Figure 2:**
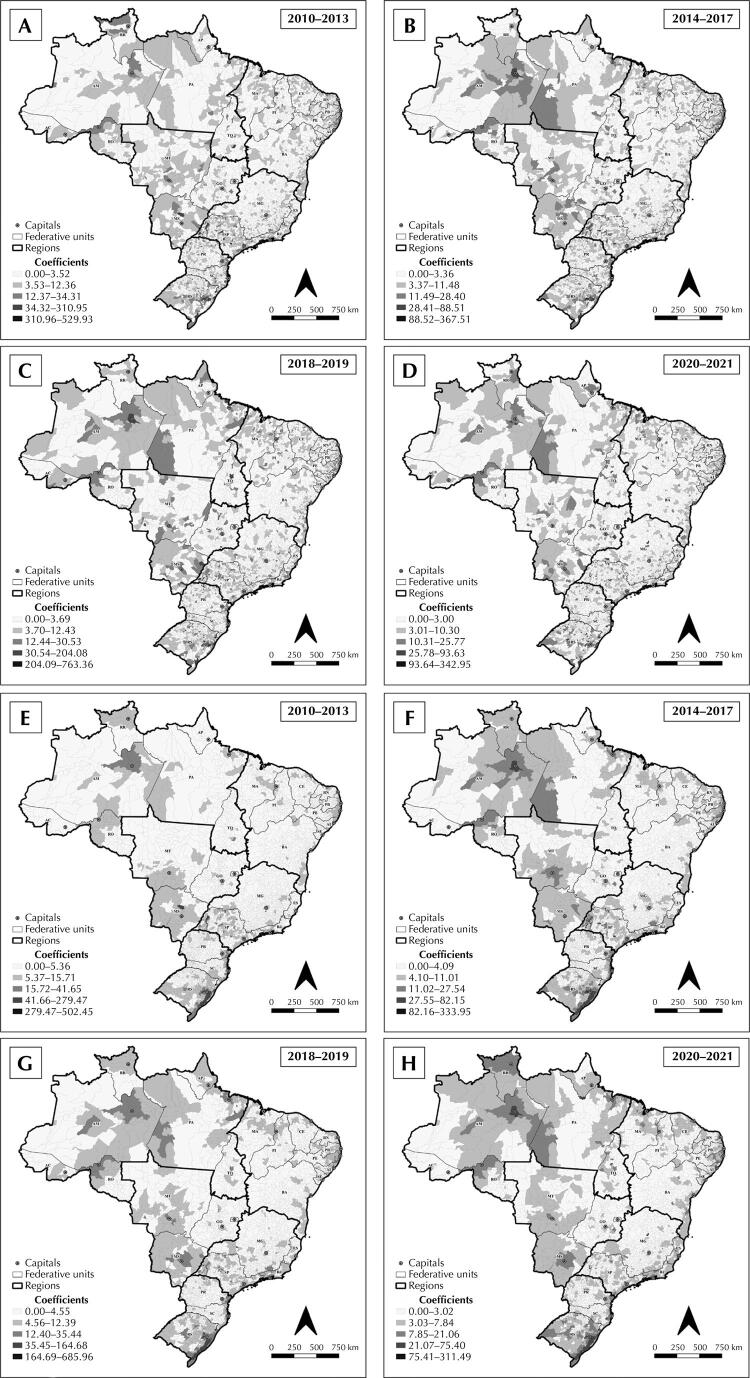
Spatial distribution of the crude (A, B, C, and D) and smoothed (E, F, G, and H) coefficient for the incidence of tuberculosis-HIV coinfection among people aged 18 to 59 years, per 100,000 inhabitants, in Brazilian municipalities, from 2010 to 2021.

In the South, cases of tuberculosis-HIV coinfection predominated on the coast, in the surroundings of the capitals and, above all, in Rio Grande do Sul. In the Southeast, São Paulo and Rio de Janeiro had the highest coefficients. In the Northeast, it was higher on the coast of Sergipe, Alagoas, Pernambuco, and Paraíba. In the Midwest, Mato Grosso do Sul and Mato Grosso stood out. In the North, the highest coefficients were in Roraima, Pará, and Amazonas (Figure 2).

**Figure 3:**
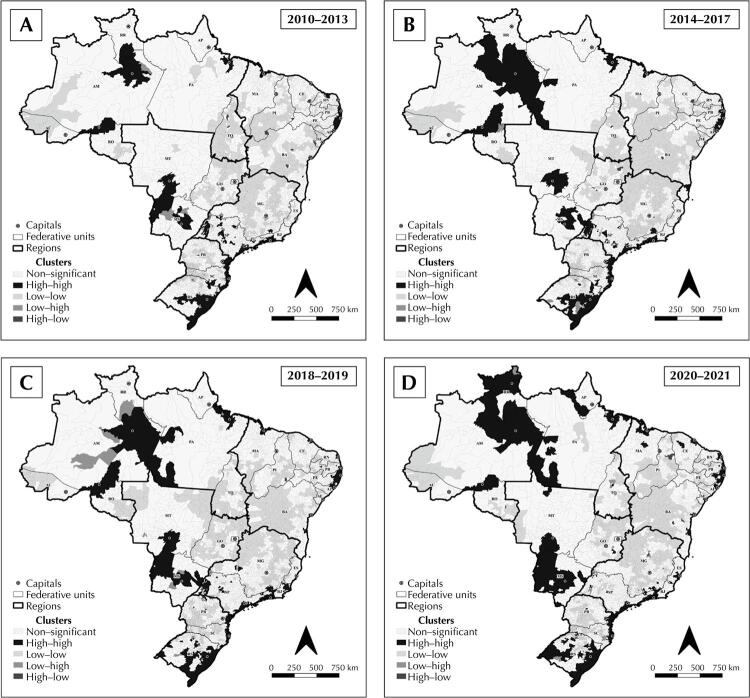
Spatial distribution of the clusters from the univariate analysis of the smoothed coefficient for the incidence of tuberculosis-HIV coinfection among people aged 18 to 59 years, per 100,000 mil inhabitants, in Brazilian municipalities, from 2010 to 2021.

The univariate analysis using Moran’s global statistics confirmed the existence of spatial dependence (I_MG_: +0.164 and p = 0.001 for 2010–2013; I_MG_: +0.220 and p = 0.001 for 2014–2017; I_MG_: +0.094 and p = 0.002 for 2018–2019; and I_MG_: +0.266 and p = 0.001 for 2020–2021). Thus, a local analysis was performed, which showed clusters of type HH and LL, as well as transition groups of type LH and HL, as shown in Figure 3.

Over the years, the spatial dependence of tuberculosis-HIV coinfection in Brazilian municipalities varied little. Clusters of areas and neighbors with high coefficients were evidenced in the regions close to the capitals of Amazonas, Roraima, Tocantins, Rondônia, and Pará. In the Northeast region, these groupings were observed in the host cities of Ceará and Maranhão, as well as in the coastal states (Figure 3).

In the Midwest, Mato Grosso and Mato Grosso do Sul stood out, with HH-type clusters in the capitals and in the border region between the states. In the South and Southeast, clusters of municipalities and neighbors with high coefficients were noted, especially on the coast. Clusters were also observed on the border between São Paulo and Mato Grosso do Sul and, likewise, on the border between Rio Grande do Sul and other South American countries (Figure 3).

On the other hand, LL-type clusters were evidenced in Acre and its border with Amazonas, and in Rondônia and its border with Mato Grosso. The states of Goiás, Tocantins, Piauí, Bahia, Paraná, and Santa Catarina also showed significant pockets, formed by municipalities and neighbors with low coincidence coefficients of coinfection during the analyzed period (Figure 3).

**Figure 4:**
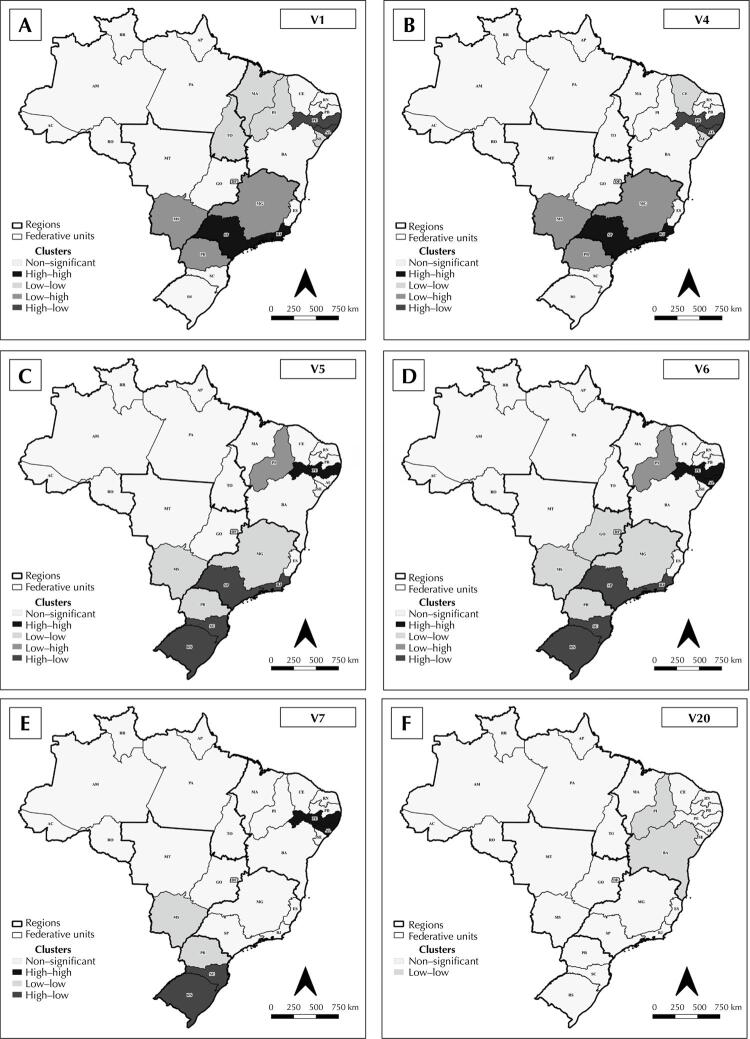
Spatial distribution of the clusters from the bivariate analysis of the crude coefficient for the incidence of tuberculosis-HIV coinfection among people aged 18 to 59 years, per 100,000 inhabitants, and of the development index (V1a, V4b, V5c, V6d, V7e, and V20f) of the Brazilian federative units, from 2010 to 2021.

^a^ Human development index.

^b^ Human development index – education.

^c^ Percentage of people vulnerable to poverty.

^d^ Percentage of poor people.

^e^ Gini index.

^f^ Aids incidence rate.

Moran’s bivariate analysis proved the existence of correlation for six variables, namely: V1 (I_MG_: +0.190 and p = 0.034); V4 (I_MG_: +0.201 and p = 0.034); V5 (I_MG_: −0.211 and p = 0.021); V6 (I_MG_: −0.195 and p = 0.026); V7 (I_MG_: −0.293 and p = 0.002); and V20 (I_MG_: +0.272 and p = 0.014). A higher occurrence of tuberculosis-HIV coinfection was observed in states with lower Gini coefficients, high aids rates, a low proportion of poor or those vulnerable to poverty, and higher development indices (Figure 4).

## DISCUSSION

The geospatialization of tuberculosis-HIV coinfection revealed heterogeneity among the FUs. Higher coefficients were found in municipalities in the South, Midwest, and North regions, with important high-risk clusters in the capitals, on borders, and on the coast. The incidence coefficients decreased in Brazil and its regions in 2020 and 2021. Moreover, coinfection showed a direct association with development indices and aids rates, and an indirect association with the percentage of poverty.

To understand the interaction between tuberculosis and HIV, we can resort to the syndemic theory. Syndemic is understood as the overlapping of certain health problems or diseases that affect the population and that can be aggravated by social, biological, and environmental factors^22,23^. This intersection increases the burden of consequences of the coexistence of these conditions, leading to repercussions in different spheres of the affected person’s life^22^.

Tuberculosis is one of the most common infections among people living with HIV, and the chances of death are progressively increased in relation to the increase in immunosuppression^1,24^. Thus, coinfection is related to the use of antiretroviral therapy (ART), and it can be assumed that its inappropriate use, whether due to factors related to the lack of supply or access in a timely manner and/or lack of adherence, may be associated with the incidence of tuberculosis-HIV^25^.

The occurrence of coinfection results from the relationship between three dimensions encompassed by vulnerability: individual, which encompasses the provision of information and the adoption of prevention and care practices; social, which includes situations related to experiences in the life cycle, such as interpersonal relationships, socioeconomic issues, and educational level; and programmatic, which permeates the supply of and access to health, social assistance, education, and culture services and actions^21,26,27^.

People living with HIV, who commonly experience stigma and prejudice, are in a state of vulnerability making them more susceptible to tuberculosis, which is related to socioeconomic conditions, accentuated in unequal territories^28,29^. These disparities were reflected in the spatial heterogeneity of coinfection in Brazil, as observed in Ethiopia, Kenya, and Uganda, countries that also have a high burden of tuberculosis-HIV^30^.

This behavior can be attributed to two aspects. The first is related to infrastructure, since places with insufficient development tend to have a higher occurrence of coinfection, such as in the North, Northeast, and Midwest regions^32^. The second is due to the epidemiology of infections, since clusters of tuberculosis-HIV can occur in areas with high HIV prevalence and/or tuberculosis incidence, such as in the South and Southeast regions^33^.

Specifically in Mato Grosso, Mato Grosso do Sul, and Rio Grande do Sul, where high-risk clusters were evidenced, a study pointed to a higher incidence of HIV^34^. The survival of people living with HIV has increased substantially with the availability and adherence to ART, contributing to the increase in the prevalence of the condition^36^. Nevertheless, the occurrence of coinfection persisted in these states, prompting a warning to the authorities.

With the cascade of continuous care, reducing the immune impairment of people with HIV and, thus, preventing the emergence of opportunistic infections is possible^37^. That said, the results suggest failures in access to and/or adherence to ART, in addition to the possibility of late diagnosis, at which point the person may already have aids. In Brazil, the diagnosis with the first CD4+ lymphocyte count below 200 cells/ml has been above 26% since 2019^38^.

Late diagnosis is linked to individual issues, such as lack of resources for transportation, social status, or distance between the home and the facility; and/or collective issues, such as stigma about HIV, fear of disclosing the diagnosis, lack of a support network, failure to articulate the network, and political and legal obstacles that weaken access to care^39,40^. This demonstrates the need for policies and actions capable of reaching all these singularities.

In addition, pockets of risk for tuberculosis-HIV coinfection were evidenced in the capitals and borders. Host cities tend to have high rates of tuberculosis, since higher population density and migratory flow act as important factors for community transmission^33^. Intra- and inter-country border regions, in turn, represent predictors for the high occurrence of infections, notably due to the greater presence of socioeconomic inequities^41,42^.

Especially in Amazonas and Acre, where high-burden clusters have been observed, geographical barriers should be considered, which may culminate in late access to HIV diagnosis or difficulty in following up people living with the virus^43^. A study conducted in the North region, the largest in territorial terms, showed that the coverage of primary health care services predominates in the host cities of the states^44^.

Hence, we can understand the correlations regarding the occurrence of tuberculosis-HIV coinfection in the states with the best indicators. These findings are contradictory to tuberculosis, which is usually associated with insufficient income, employment, and housing factors^45^. On the other hand, the behavior of coinfection is similar to that of HIV, since high rates are found in places with better living conditions and infrastructure, such as state capitals^46^.

In this sense, the importance of apprehending coinfection beyond the individual is recognized, considering issues related to the social and programmatic. Thus, although the control of tuberculosis-HIV progressed globally, it still shows gaps to mitigate this syndemic, especially due to the need for intersectoral integration to combat the obstacles that hinder access to services and continuity of care for people living with HIV.

In addition, the incidence of tuberculosis-HIV coinfection decreased in 2020 and 2021, possibly due to the effect of covid-19 on the health care network. Globally, the reallocation of resources in response to the pandemic has hampered care delivery since systems have focused on responding to this emergency. Moreover, access to services is limited due to fear of the virus, distancing measures, and reduced activity time in establishments^47^.

During the pandemic, active search for tuberculosis cases and contacts was discontinued^48^, while HIV services were restricted^49^. These factors have hindered the initiation and continuity of follow-up of people with tuberculosis and/or HIV. As a result, underdiagnosing and/or underreporting of these infections increased^20,50^ as well as coinfection, which, in this study, had a 24% reduction in incidence compared with the previous biennium.

Thus, we need to strengthen the articulation between tuberculosis and HIV programs, to promote strategies to combat inequities and, consequently, reduce the coinfection burden in the country^50^. In addition, expanding the coverage of care strategies for the diagnosis and prevention of tuberculosis disease, as well as for screening and treatment of tuberculosis infection, both in people living with HIV and in the general population, is essential.

The potential contribution of this study is anchored since it highlights areas of high incidence of tuberculosis-HIV coinfection and its relationship with health and socioeconomic indicators. Such findings can help the elaboration, implementation, and/or strengthening of intersectoral policies that seek to transform reality to achieve the 2030 goals, based on the construction of sensitive indicators and the creation of strategies unique to Brazilian territories.

Note that the results should be interpreted considering limitations. The unavailability of updated contextual indicators for the municipalities restricted the analysis and interpretation of the data. This is due to the impossibility of identifying differences that may exist between municipalities, including variations in population distribution, access to health services, and other determinants of the health-disease process, when working with aggregated data at the state level.

Despite this and the problems associated with the use of secondary data, such as filling errors, incompleteness, and/or differential underreporting, state-level analysis plays an important role in decision-making, since it allows for a comprehensive and contextual understanding of disparities in a given region. This approach is fundamental to define priorities, since they generate a broad view of territorial inequalities.

In summary, the heterogeneous pattern of the distribution of tuberculosis-HIV coinfection in Brazil was evidenced, with disparities between municipalities and little change in spatial behavior over the period. High coefficients were observed in Rio Grande do Sul, Mato Grosso do Sul, and Amazonas, as well as important high-risk clusters in areas close to state capitals, intra- and inter-country border regions, and coastal cities.

A higher occurrence of coinfection was also noted in states with high aids rates, a low proportion of poor or those vulnerable to poverty, and higher development indices. However, the associations are not generalizable and should be interpreted cautiously, since states with low infrastructure also had high tuberculosis-HIV coefficients, possibly due to personal, territorial, and/or socio-sanitary aspects linked to infections.
