# Supplementary material for: Clusters of heterogeneity of tuberculosis-HIV coinfection in Brazil: a geospatial study
Source: Rev Saude Publica. 2024 Mar 27;58:10. doi: 10.11606/s1518-8787.2024058005531 (PMC11037911; doi:10.11606/s1518-8787.2024058005531)
Supplement: Translation

# Translation: Clusters da heterogeneidade da coinfecção tuberculose-HIV no Brasil: um estudo geoespacial

**Authors:** Lucas Vinícius de Lima, Gabriel Pavinati, Pedro Augusto Bossonario, Aline Aparecida Monroe, Daniele Maria Pelissari, Kleydson Bonfim Andrade Alves, Gabriela Tavares Magnabosco

**Affiliations:** I Brasil Universidade Estadual de Maringá. Programa de Pós-Graduação em Enfermagem. Maringá, PR, Brasil; II Brasil Universidade de São Paulo. Programa de Pós-Graduação Enfermagem em Saúde Pública. Ribeirão Preto, SP, Brasil; III Brasil Ministério da Saúde. Departamento de HIV/aids, Tuberculose, Hepatites Virais e Infecções Sexualmente Transmissíveis. Brasília, DF, Brasil; IV Brasil Organização Pan-Americana da Saúde. Departamento de Doenças Transmissíveis e Determinantes Ambientais da Saúde. Brasília, DF, Brasil

**Keywords:** HIV, Tuberculose, Coinfecção, Análise Espacial

## Abstract

**OBJETIVO:**

Analisar a geoespacialização da coinfecção tuberculose-HIV no Brasil, de 2010 a 2021, e a correlação com indicadores socioeconômicos, habitacionais e sanitários.

**MÉTODOS:**

Estudo ecológico dos municípios e estados brasileiros, com dados dos sistemas de informação do HIV e da tuberculose, previamente relacionados pelo Ministério da Saúde. Foram calculados os coeficientes brutos e suavizados pelo método bayesiano empírico local de incidência da coinfecção, por 100 mil habitantes, na população entre 18 e 59 anos. Empregaram-se os índices de Moran univariado (identificação de *clusters*) e bivariado (correlação com 20 indicadores).

**RESULTADOS:**

Foram registrados 122.223 casos de coinfecção no Brasil, de 2010 a 2021, com coeficiente médio de 8,30/100 mil. As regiões Sul (11,44/100 mil) e Norte (9,93/100 mil) concentraram a maior carga das infecções. Houve queda dos coeficientes no Brasil, em todas as regiões, nos anos de covid-19 (2020 e 2021). Os maiores coeficientes foram visualizados nos municípios do Rio Grande do Sul, do Mato Grosso do Sul e do Amazonas, com aglomerados alto-alto nas capitais, em regiões de fronteira e no litoral do país. Os municípios pertencentes aos estados de Minas Gerais, da Bahia, do Paraná e do Piauí apresentaram *clusters* baixo-baixo. Houve correlação direta com os índices de desenvolvimento humano e as taxas de aids, bem como indireta com a proporção de pobres ou vulneráveis à pobreza e o índice de Gini.

**CONCLUSÕES:**

A análise espacial da coinfecção tuberculose-HIV demonstrou heterogeneidade no território brasileiro e comportamento constante ao longo do período, revelando *clusters* com municípios de alta carga, principalmente nos grandes centros urbanos e nos estados com ocorrência elevada do HIV e/ou da tuberculose. Esses achados, além de trazerem um alerta para os efeitos da pandemia da covid-19, podem incorporar o planejamento estratégico para o controle da coinfecção, visando à eliminação dessas infecções como problemas de saúde pública até 2030.

## INTRODUÇÃO

A tuberculose e a infecção pelo vírus da imunodeficiência humana (*human immunodeficiency virus* – HIV) persistem como desafios à saúde pública, principalmente para os sistemas e os serviços de saúde, dada a complexidade decorrente da possível sobreposição dessas condições, bem como a existência de aspectos estruturais, organizacionais, socioeconômicos, culturais e comportamentais que tangenciam sua ocorrência^1^.

Há uma forte relação entre a tuberculose e o HIV, em especial pela maior suscetibilidade das pessoas acometidas pelo vírus para o desenvolvimento da forma ativa e dos desfechos desfavoráveis da tuberculose^1,3,4^, tornando-as um grupo prioritário às ações de prevenção, tratamento e controle. Em 2021, foram registrados 6,4 milhões de casos de tuberculose a nível mundial, sendo que 6,7% foram em pessoas vivendo com HIV e, desses, 187 mil tiveram como desfecho o óbito, reiterando a alta mortalidade dessas infecções^2^.

Findar as epidemias de tuberculose e HIV, bem como a concomitância das infecções, figura entre os Objetivos do Desenvolvimento Sustentável (ODS), firmados pela Organização das Nações Unidas (ONU)^1,5^. Esses objetivos são ainda mais importantes nos 30 países de alta carga de coinfecção listados pela Organização Mundial da Saúde (OMS), entre os quais se destaca o Brasil, que teve proporção de 10,3% de HIV entre os casos de tuberculose em 2019^1,2,6^.

Dadas as dimensões continentais do país, existem discrepâncias no comportamento da coinfecção entre os territórios brasileiros, devido a fatores sociais e programáticos^6^. Reconhece-se que o agravo atinge, sobretudo, pessoas que vivenciam situações de vulnerabilidade, experienciando condições precárias de vida, dispondo de práticas de risco à saúde e/ou prescindindo de recursos para o acesso às estratégias de prevenção, diagnóstico e tratamento do HIV e da tuberculose^7,9^.

Apesar da elevada morbimortalidade da coinfecção tuberculose-HIV, há certa dificuldade na realização de estudos acerca do cenário epidemiológico no Brasil^7,8,10,11^, possivelmente pela fragilidade do registro da dupla infecção, que requer o relacionamento entre as bases de dados de ambas para a qualificação da notificação^6^. Avulta-se, então, a necessidade de estudos acerca da influência do território geopolítico e social na ocorrência da coinfecção.

Nessa direção, os estudos espaciais têm sido essenciais à vigilância das medidas de incidência e morbimortalidade^7,12^, sobretudo na interface com as condições infectocontagiosas que, historicamente, se relacionam às disparidades do território brasileiro^13^. Isso posto, este estudo teve como objetivo analisar a geoespacialização da incidência da coinfecção tuberculose-HIV no Brasil, de 2010 a 2021, e a correlação com indicadores socioeconômicos, habitacionais e sanitários.

## MÉTODOS

Estudo ecológico geoespacial, cujas unidades analíticas foram as regiões geográficas, as unidades federativas (UF) e os municípios brasileiros. Em sua divisão político-administrativa, o Brasil contempla 5.568 municípios – mais o Distrito Federal e o Distrito Estadual de Fernando de Noronha –, que se organizam em 27 UF. Essas, por sua vez, estão dispostas em cinco regiões geográficas: Norte, Nordeste, Sul, Sudeste e Centro-Oeste^14^.

A vigilância do HIV e da tuberculose orienta-se por programas vinculados à Secretaria de Vigilância em Saúde e Ambiente do Ministério da Saúde (SVSA/MS). A partir das recomendações nacionais, as UF e os municípios se organizam para a oferta de ações e serviços voltados ao manejo e ao controle da tuberculose e do HIV. As informações em saúde, por sua vez, são centralizadas nos sistemas de notificação e acompanhamento de base nacional.

Para delimitar os casos de coinfecção tuberculose-HIV, foram considerados os dados do Sistema de Informação de Agravos de Notificação (Sinan), do Sistema de Controle de Exames Laboratoriais da Rede Nacional de Contagem de Linfócitos CD4+/CD8+ e Carga Viral do HIV (Siscel), do Sistema de Controle Logístico de Medicamentos (Siclom) e do Sistema de Informação sobre Mortalidade (SIM), relacionados de forma probabilística no *software* Reclink®.

O banco de dados, conforme descrição metodológica no boletim epidemiológico da coinfecção tuberculose-HIV publicado em 2021^6^, foi solicitado ao Ministério da Saúde, via Sistema Eletrônico de Informações ao Cidadão (e-SIC), sob protocolo nº 25072.039887/2022-27. Foram realizados três relacionamentos probabilísticos: o primeiro entre as bases do HIV; o segundo entre as bases da tuberculose; e o terceiro entre os produtos das duas primeiras etapas^6^.

Os dados populacionais do estudo de estimativas foram acessados no Departamento de Informática do Sistema Único de Saúde (Datasus)^15^. Ademais, os indicadores socioeconômicos, habitacionais e sanitários, oriundos do Sistema Nacional de Informações sobre Saneamento (SNIS), da Pesquisa Nacional por Amostra de Domicílios (PNAD) e do Datasus, referentes a 2017, foram consultados no Atlas do Desenvolvimento Humano^16^ (Quadro).

**Quadro:** Indicadores de desenvolvimento considerados para a correlação com os coeficientes brutos de incidência da coinfecção tuberculose-HIV das unidades federadas do Brasil.

Indicador	Código	Fonte
Socioeconômico		
Índice de desenvolvimento humano	V1	PNAD (2017)
Índice de desenvolvimento humano – renda	V2	PNAD (2017)
Índice de desenvolvimento humano – longevidade	V3	PNAD (2017)
Índice de desenvolvimento humano – educação	V4	PNAD (2017)
Porcentagem de vulneráveis à pobreza	V5	PNAD (2017)
Porcentagem de pobres	V6	PNAD (2017)
Índice de Gini	V7	PNAD (2017)
Renda *per capita*	V8	PNAD (2017)
Porcentagem de 18 anos ou mais de idade com ensino fundamental completo	V9	PNAD (2017)
Taxa de analfabetismo de 18 anos ou mais de idade	V10	PNAD (2017)
Média de anos de estudo	V11	PNAD (2017)
Habitacional		
Porcentagem de domicílios ligados à rede de abastecimento de água	V12	SNIS (2017)
Porcentagem de domicílios ligados à rede de esgotamento sanitário	V13	SNIS (2017)
Existência de coleta seletiva	V14	SNIS (2017)
Porcentagem de internações relacionadas ao saneamento ambiental inadequado	V15	Datasus (2017
Sanitário		
Esperança de vida ao nascer	V16	PNAD (2017)
Porcentagem de internações por condições sensíveis à atenção primária	V17	Datasus (2017)
Porcentagem de pessoas cobertas por planos de saúde suplementar	V18	Datasus (2017)
Taxa bruta de mortalidade	V19	Datasus (2017)
Taxa de incidência de aids	V20	Datasus (2017)

PNAD: Pesquisa Nacional por Amostra de Domicílios; SNIS: Sistema Nacional de Informações sobre Saneamento; Datasus: Departamento de Informática do Sistema Único de Saúde.

Definiu-se como casos de coinfecção as pessoas cujo registro de tuberculose no Sinan (notificadas como “caso novo”), independentemente da forma clínica, apresentava a variável “HIV” assinalada como “positivo” ou a variável “agravo aids” assinalada como “sim”; ou aquelas notificadas por tuberculose no Sinan ou SIM sem o preenchimento dessas variáveis, mas que possuíam diagnóstico nas bases do HIV, que apresentavam algum resultado no Siscel ou que tinham dispensação no Siclom^6^.

Foram incluídas pessoas de 18 a 59 anos, uma vez que correspondem à maior parte dos casos de coinfecção registrados no país (± 91,88%)^6^. Considerou-se, ainda, a variável referente ao município de residência. Contudo, ressalta-se que 68 pessoas tiveram essa informação ignorada no momento da notificação. Assim, o georreferenciamento desses casos se deu pelo município de notificação, representando uma variável *proxy* da residência.

O recorte temporal de 2010 a 2021 contemplou os períodos: anterior à incorporação do teste rápido molecular para tuberculose (2010–2013); anterior à publicação do protocolo de vigilância da tuberculose infecção (2014–2017); anterior à pandemia da covid-19 (2018–2019); e concomitante à covid-19 (2020–2021). Essas intervenções podem ter influenciado na prevenção, detecção e/ou notificação da tuberculose em pessoas com HIV e, portanto, modificado o comportamento espacial.

Os dados foram tabulados de acordo com os quadriênios e biênios supracitados. Inicialmente, para a distribuição espacial dos casos de coinfecção tuberculose-HIV, foram calculados os coeficientes brutos de incidência para cada município, UF e região do país, a partir da razão dos casos novos pela população residente, no mesmo período, idade e localidade. O resultado foi multiplicado por 100 mil habitantes.

Dada a existência de municípios pouco populosos, a observação de casos poderia levar à baixa representatividade dos coeficientes, ocasionando distorção e alta variabilidade. Sendo assim, foi procedida a suavização pelo método bayesiano empírico local, visando à redução das instabilidades ao gerar coeficientes pela ponderação dos valores das fronteiras, definidas pela contiguidade do tipo *queen* de primeira ordem, a fim de restringir a flutuação aleatória^17^.

Com isso, foram construídos mapas para a distribuição espacial, organizados a partir das quebras naturais em uma escala de tons cinzas com cores claras e escuras, representando os coeficientes mínimos e máximos, respectivamente. Essa classificação garantiu a homogeneidade e a heterogeneidade dos dados, ao realizar a “quebra” dos intervalos, minimizando sua variação em um mesmo grupo e maximizando a variação dos valores entre os grupos^18^.

Empregou-se a estatística de Moran nos coeficientes suavizados, subdividida em índice de Moran global (I_MG_) e local (I_ML_). Foi calculado o I_MG_ univariado com o respectivo teste de pseudossignificância, com 999 permutações (p < 0,05). O I_MG_ testa a hipótese de dependência espacial, fornecendo uma medida que varia de -1,00 a +1,00. Valores próximos a zero indicam aleatoriedade e valores próximos a um indicam autocorrelação espacial direta (+) ou inversa (-)^19^.

Uma vez comprovada a significância do I_MG_, foi calculado o I_ML_ univariado, um tipo de indicador local de associação espacial para identificar agrupamentos que influenciam no I_MG_, sendo eles: alto-alto (AA), áreas e vizinhos com altos coeficientes; baixo-baixo (BB), áreas e vizinhos com baixos coeficientes; baixo-alto (BA), áreas com baixos e vizinhos com altos coeficientes; alto-baixo (AB), áreas com altos e vizinhos com baixos coeficientes; e não significante (NS), áreas sem tendência espacial^20^.

Para a associação dos coeficientes de incidência da coinfecção tuberculose-HIV com os indicadores dispostos no Quadro, utilizaram-se dados agregados a nível estadual, dada a indisponibilidade de indicadores recentes para os municípios do Brasil na data de realização deste estudo. Dessa forma, as médias aritméticas dos coeficientes brutos de incidência de cada UF foram consideradas como a variável dependente e os indicadores como as variáveis independentes.

Esses coeficientes médios foram logaritmizados para aproximá-los de uma distribuição normal. Então, construiu-se uma matriz de correlação de Spearman para a análise bivariada, na qual as variáveis foram incapazes de apresentar associação significativa (p > 0,50), impossibilitando a construção de um modelo de regressão. Por conta disso, empregou-se a análise bivariada de Moran, que busca verificar a associação entre duas variáveis com base nos valores nas regiões vizinhas^20^.

Na análise bivariada de Moran, os aglomerados foram classificados em: AA, áreas com altos coeficientes e indicadores; BB, áreas com baixos coeficientes e indicadores; BA, áreas com baixos coeficientes e altos indicadores; AB, áreas com altos coeficientes e baixos indicadores; e NS, áreas sem associação^20^. As análises foram realizadas em planilha do Microsoft Excel® 2016 e nos *softwares* GeoDa® (versão 1.20.0) e QGIS® (versão 2.36.3).

Com intuito de ampliar o aprofundamento do estudo, recorreu-se ao referencial da vulnerabilidade, que dispõe de três planos indissociáveis e interdependentes, representados por aspectos individuais, sociais e programáticos, que influenciam no processo saúde-doença. Desse modo, expande-se o conceito de risco, assumindo que o adoecimento leva em consideração situações associadas aos contextos socioeconômico, institucional e geopolítico^21^.

De acordo com as diretrizes éticas, preconizadas pela Resolução nº 466/2012 do Conselho Nacional de Saúde, esta pesquisa foi aprovada pelo Comitê de Ética em Pesquisa da Universidade Estadual de Maringá, sob parecer nº 5.721.740/2022 e certificado de apresentação para apreciação ética nº 63981922.6.0000.0104. Solicitou-se a dispensa do termo de consentimento livre e esclarecido pelo uso de dados secundários.

## RESULTADOS

Foram notificados 122.223 casos de coinfecção tuberculose-HIV no Brasil, entre pessoas de 18 a 59 anos, de 2010 a 2021, com coeficiente médio de 8,30/100 mil. As regiões Sul e Norte concentraram o maior quantitativo de casos. No Norte, houve aumento entre 2014 e 2017, ao passo que o Sul e o Sudeste mostraram redução ao longo dos anos. Foi evidenciada queda da incidência da coinfecção no país e suas macrorregiões entre 2020 e 2021 (Figura 1).

**Figura 1:** Coeficientes brutos de incidência da coinfecção tuberculose-HIV entre pessoas de 18 a 59 anos, por 100 mil habitantes, nas regiões do Brasil de 2010 a 2021.

No Sul, os casos de coinfecção tuberculose-HIV predominaram no litoral, no entorno das capitais e, sobretudo, no Rio Grande do Sul. No Sudeste, São Paulo e Rio de Janeiro tiveram os municípios com os coeficientes mais elevados. No Nordeste, viu-se maior ocorrência no litoral de Sergipe, do Alagoas, de Pernambuco e da Paraíba. No Centro-Oeste, destacaram-se o Mato Grosso do Sul e o Mato Grosso. Já no Norte, os maiores coeficientes foram em Roraima, no Pará e no Amazonas (Figura 2).

**Figura 2:** Distribuição espacial dos coeficientes brutos (A, B, C e D) e suavizados (E, F, G e H) de incidência da coinfecção tuberculose-HIV entre pessoas de 18 a 59 anos, por 100 mil habitantes, nos municípios do Brasil de 2010 a 2021.

A análise univariada pela estatística global de Moran comprovou a existência de dependência espacial (I_MG_: +0,164 e p = 0,001 para 2010–2013; I_MG_: +0,220 e p = 0,001 para 2014–2017; I_MG_: +0,094 e p = 0,002 para 2018–2019; e I_MG_: +0,266 e p = 0,001 para 2020–2021). Dessa forma, procedeu-se à análise local, que evidenciou aglomerados do tipo AA e BB, bem como grupos de transição do tipo BA e AB, conforme apresentado na Figura 3.

**Figura 3:** Distribuição espacial dos clusters da análise univariada dos coeficientes suavizados de incidência da coinfecção tuberculose-HIV entre pessoas de 18 a 59 anos, por 100 mil habitantes, nos municípios do Brasil de 2010 a 2021.

Ao longo dos anos, percebeu-se pouca variação da dependência espacial da coinfecção tuberculose-HIV nos municípios brasileiros. Foram evidenciados *clusters* de áreas e vizinhos com altos coeficientes nas regiões próximas às capitais do Amazonas, de Roraima, do Tocantins, de Rondônia e do Pará. Na região Nordeste, visualizaram-se esses agrupamentos nas cidades-sede do Ceará e do Maranhão, bem como nos estados litorâneos (Figura 3).

No Centro-Oeste, destacaram-se o Mato Grosso e o Mato Grosso do Sul, com *clusters* do tipo AA nas capitais e na região de fronteira entre os estados. No Sul e no Sudeste, foram notados agrupamentos de municípios e vizinhos com altos coeficientes, especialmente no litoral. Perceberam-se, ainda, *clusters* na fronteira de São Paulo com Mato Grosso do Sul e, do mesmo modo, no limite do Rio Grande do Sul com outros países sul-americanos (Figura 3).

Em contrapartida, foram evidenciados agrupamentos do tipo BB no Acre e sua divisa com o Amazonas e, igualmente, em Rondônia e sua fronteira com o Mato Grosso. Os estados de Goiás, do Tocantins, do Piauí, da Bahia, do Paraná e de Santa Catarina também apresentaram bolsões significativos, formados por municípios e vizinhos com baixos coeficientes de incidência da coinfecção no decorrer do período analisado (Figura 3).

A análise bivariada de Moran comprovou a existência de correlação para seis variáveis, sendo elas: V1 (I_MG_: +0,190 e p = 0,034); V4 (I_MG_: +0,201 e p = 0,034); V5 (I_MG_: -0,211 e p = 0,021); V6 (I_MG_: -0,195 e p = 0,026); V7 (I_MG_: -0,293 e p = 0,002); e V20 (I_MG_: +0,272 e p = 0,014). Visualizou-se maior ocorrência da coinfecção tuberculose-HIV em estados com menores coeficientes de Gini, altas taxas de aids, baixa proporção de pobres ou vulneráveis à pobreza e maiores índices de desenvolvimento (Figura 4).

**Figura 4:** Distribuição espacial dos clusters da análise bivariada dos coeficientes brutos de incidência da coinfecção tuberculose-HIV entre pessoas de 18 a 59 anos, por 100 mil habitantes, e dos indicadores de desenvolvimento (V1a, V4b, V5c, V6d, V7e e V20f) nas unidades federadas do Brasil de 2010 a 2021.

^a^ Índice de desenvolvimento humano.

^b^ Índice de desenvolvimento humano – educação.

^c^ Porcentagem de vulneráveis à pobreza.

^d^ Porcentagem de pobres.

^e^ Índice de Gini.

^f^ Taxa de incidência de aids.

## DISCUSSÃO

A geoespacialização da coinfecção tuberculose-HIV revelou heterogeneidade entre as UF. Foram evidenciados maiores coeficientes em municípios das regiões Sul, Centro-Oeste e Norte, com importantes aglomerados de alto risco nas capitais, em fronteiras e no litoral. Observou-se redução nos coeficientes de incidência no Brasil e suas regiões em 2020 e 2021. Houve, ainda, associação direta da coinfecção com os índices de desenvolvimento e a taxa de aids e indireta com a porcentagem de pobreza.

Para compreender a interação entre tuberculose e HIV, pode-se recorrer à teoria sindêmica. Entende-se como sindemia a sobreposição de determinados agravos ou doenças que acometem a população e que podem ser agravados por fatores sociais, biológicos e ambientais^22,23^. Esse entrecruzamento eleva a carga de consequências da coexistência dessas condições, levando a repercussões em diferentes esferas na vida da pessoa acometida^22^.

A tuberculose é uma das infecções mais comuns entre as pessoas vivendo com HIV, tendo as chances de morte aumentadas progressivamente em relação ao crescimento da imunossupressão^1,24^. Assim, existe uma relação entre a coinfecção e a utilização da terapia antirretroviral (TARV), podendo-se presumir que o seu uso inadequado, seja por fatores referentes à ausência de oferta ou de acesso em tempo oportuno e/ou à falta de adesão, pode estar associado à incidência de tuberculose-HIV^25^.

A ocorrência da coinfecção resulta da relação entre três dimensões abarcadas pela vulnerabilidade: individual, que engloba a disposição de informações e a adoção de práticas de prevenção e cuidado; social, que inclui situações referentes às vivências no ciclo de vida, como relações interpessoais, questões socioeconômicas e nível educacional; e programática, que perpassa a oferta e o acesso aos serviços e às ações de saúde, assistência social, educação e cultura^21,26,27^.

Reconhece-se que há um estado de vulnerabilização no qual as pessoas vivendo com HIV, que comumente vivenciam estigma e preconceito, tornam-se mais suscetíveis à tuberculose, estando relacionada às condições socioeconômicas, o que se acentua em territórios desiguais^28,29^. Essas disparidades refletiram na heterogeneidade espacial da coinfecção no Brasil, assim como observado na Etiópia, no Quênia e na Uganda, países também de alta carga de tuberculose-HIV^30^.

Esse comportamento pode ser atribuído a dois aspectos. O primeiro diz respeito à infraestrutura, visto que locais com desenvolvimento insuficiente tendem a apresentar maior ocorrência da coinfecção, como nas regiões Norte, Nordeste e Centro-Oeste^32^. O segundo decorre da epidemiologia das infecções, pois aglomerados de tuberculose-HIV podem ocorrer em áreas de elevada prevalência do HIV e/ou incidência da tuberculose, como nas regiões Sul e Sudeste^33^.

Especificamente no Mato Grosso, no Mato Grosso do Sul e no Rio Grande do Sul, onde foram evidenciados *clusters* de alto risco, estudo apontou para maior incidência do HIV^34^. Sabe-se que a sobrevida das pessoas vivendo com HIV aumentou substancialmente com a disponibilidade e a adesão à TARV, contribuindo para o aumento da prevalência da condição^36^. Não obstante, a ocorrência da coinfecção persistiu nesses estados, figurando um alerta às autoridades.

Por meio da cascata de cuidado contínuo, pode-se reduzir o comprometimento imunológico das pessoas com HIV e, dessa forma, prevenir o surgimento de infecções oportunistas^37^. Posto isso, os resultados sugerem falhas no acesso e/ou na adesão à TARV, além da possibilidade de diagnóstico tardio, momento em que a pessoa já pode se encontrar com a aids. No Brasil, o diagnóstico com a primeira contagem de linfócitos CD4+ abaixo de 200 céls/ml figura acima de 26% desde 2019^38^.

O diagnóstico tardio atrela-se a questões individuais, como falta de recursos para transporte, *status* social ou distância entre o domicílio e o estabelecimento; e/ou a questões coletivas, a exemplo do estigma acerca do HIV, medo da revelação do diagnóstico, ausência de rede de apoio, falha na articulação da rede e obstáculos políticos e legais que fragilizam o acesso ao cuidado^39,40^. Isso demonstra a necessidade de políticas e ações capazes de alcançar todas essas singularidades.

Ademais, foram evidenciados bolsões de risco para a coinfecção tuberculose-HIV nas capitais e nas fronteiras. As cidades-sede costumam ter altos índices de tuberculose, uma vez que a maior densidade populacional e o fluxo migratório atuam como importantes fatores para a transmissão comunitária^33^. As regiões de fronteira intra e interpaíses, por sua vez, representam preditores para a ocorrência elevada das infecções, sobretudo pela maior presença de iniquidades socioeconômicas^41,42^.

Especialmente no Amazonas e no Acre, onde foram visualizados *clusters* de alta carga, deve-se considerar as barreiras geográficas para a população, o que pode culminar no acesso tardio ao diagnóstico de HIV ou na dificuldade do seguimento das pessoas vivendo com o vírus^43^. Estudo realizado na região Norte, a maior em termos territoriais, demonstrou que a cobertura dos serviços de atenção primária à saúde predomina nas cidades-sede dos estados^44^.

Diante disso, pode-se entender as correlações em relação à ocorrência da coinfecção tuberculose-HIV nos estados com melhores indicadores. Esses achados são contraditórios à tuberculose, que, geralmente, se associa a fatores insuficientes de renda, emprego e moradia^45^. Por outro lado, o comportamento da coinfecção assemelha-se ao do HIV, uma vez que altas taxas são encontradas em locais com melhores condições de vida e infraestrutura, a exemplo das capitais^46^.

Nesse sentido, reconhece-se a importância de apreender a coinfecção para além do indivíduo, levando-se em consideração questões relativas ao social e programático. Assim, embora haja progresso global no controle da tuberculose-HIV, persistem lacunas para mitigar essa sindemia, sobretudo pela necessidade de integração intersetorial para combater os obstáculos que prejudicam o acesso aos serviços e a continuidade do cuidado das pessoas vivendo com HIV.

Ainda, assinala-se a queda da incidência da coinfecção tuberculose-HIV em 2020 e 2021, possivelmente pelo efeito que a covid-19 teve na rede de atenção à saúde. A nível mundial, a realocação de recursos em resposta à pandemia prejudicou a prestação de cuidado, pois os sistemas se centraram na resposta a essa emergência. Soma-se, também, o acesso limitado aos serviços por medo do vírus, medidas de distanciamento e redução do tempo de atividade dos estabelecimentos^47^.

Na pandemia, a busca ativa de casos e de contatos de tuberculose foi descontinuada^48^, ao passo que houve restrições nos serviços de HIV^49^. Esses fatores trouxeram obstáculos para o início e a continuidade do seguimento das pessoas com tuberculose e/ou HIV. Como reflexo, observou-se aumento do subdiagnóstico e/ou da subnotificação dessas infecções^20,50^ e, de forma semelhante, da coinfecção, que, neste estudo, teve redução de 24% na incidência em comparação ao biênio anterior.

Dessa forma, urge fortalecer a articulação entre os programas de tuberculose e HIV, com intuito de promover estratégias de enfrentamento às iniquidades e, assim, reduzir a carga de coinfecção no país^50^. Além disso, é fundamental a ampliação da cobertura assistencial das estratégias de diagnóstico e prevenção da tuberculose doença, bem como de rastreamento e tratamento da tuberculose infecção, tanto em pessoas vivendo com HIV quanto na população geral.

Assim, ancora-se o potencial de contribuição deste estudo, ao evidenciar áreas de alta incidência da coinfecção tuberculose-HIV e sua relação com indicadores sanitários e socioeconômicos. Tais achados podem auxiliar a elaboração, a implementação e/ou o fortalecimento de políticas intersetoriais que busquem transformar a realidade para o alcance das metas de 2030, a partir da construção de indicadores sensíveis e da criação de estratégias singulares aos territórios brasileiros.

Vale pontuar que os resultados devem ser interpretados à luz de limitações. A indisponibilidade de indicadores contextuais atualizados para os municípios restringiu a análise e a interpretação dos dados. Isso porque, ao se trabalhar com dados agregados no nível estadual, não é possível identificar diferenças que podem existir entre os municípios, incluindo variações na distribuição da população, acesso aos serviços de saúde e outros determinantes do processo saúde-doença.

A despeito disso e dos problemas associados ao uso de dados secundários, como erros de preenchimento, incompletudes e/ou subnotificações diferenciais, a análise a nível estadual desempenha um papel importante na tomada de decisão, pois permite uma compreensão abrangente e contextual das disparidades em uma determinada região. Essa abordagem é fundamental para a definição de prioridades, pois gera uma visão ampla sobre as desigualdades territoriais.

Em suma, evidenciou-se o padrão heterogêneo da distribuição da coinfecção tuberculose-HIV no Brasil, com disparidades entre os municípios e pouca modificação do comportamento espacial ao longo do período. Foram observados coeficientes elevados no Rio Grande do Sul, no Mato Grosso do Sul e no Amazonas, além de importantes *clusters* de alto risco em áreas nas proximidades das capitais, em regiões de fronteira intra e interpaíses e nas cidades litorâneas.

Notou-se, ainda, a maior ocorrência da coinfecção em estados com altas taxas de aids, baixa proporção de pobres ou vulneráveis à pobreza e maiores índices de desenvolvimento. Entretanto, as associações não são generalizáveis, devendo ser interpretadas cautelosamente, visto que estados com baixa infraestrutura também tiveram coeficientes altos de tuberculose-HIV, possivelmente por aspectos pessoais, territoriais e/ou sociossanitários atrelados às infecções.
